# Cell-specific occupancy dynamics between the pioneer-like factor Opa/ZIC and Ocelliless/OTX regulate early head development in embryos

**DOI:** 10.3389/fcell.2023.1126507

**Published:** 2023-03-27

**Authors:** Kelli D. Fenelon, Fan Gao, Priyanshi Borad, Shiva Abbasi, Lior Pachter, Theodora Koromila

**Affiliations:** ^1^ Department of Biology, UT Arlington, Arlington, TX, United States; ^2^ Caltech Bioinformatics Resource Center (CBRC), Caltech, Pasadena, CA, United States; ^3^ Division of Biology and Biological Engineering, California Institute of Technology, Pasadena, CA, United States; ^4^ Department of Computational Biology and Computing and Mathematical Sciences, California Institute of Technology, Pasadena, CA, United States

**Keywords:** Opa/ZIC, Oc/OTX, embryonic head development, epigenetics, *Drosophila* embryo

## Abstract

During development, embryonic patterning systems direct a set of initially uncommitted pluripotent cells to differentiate into a variety of cell types and tissues. A core network of transcription factors, such as Zelda/POU5F1, Odd-paired (Opa)/ZIC3 and Ocelliless (Oc)/OTX2, are conserved across animals. While Opa is essential for a second wave of zygotic activation after Zelda, it is unclear whether Opa drives head cell specification, in the *Drosophila* embryo. Our hypothesis is that Opa and Oc are interacting with distinct cis-regulatory regions for shaping cell fates in the embryonic head. Super-resolution microscopy and meta-analysis of single-cell RNAseq datasets show that *opa*’s and *oc*’s overlapping expression domains are dynamic in the head region, with both factors being simultaneously transcribed at the blastula stage. Additionally, analysis of single-embryo RNAseq data reveals a subgroup of Opa-bound genes to be Opa-independent in the cellularized embryo. Interrogation of these genes against Oc ChIPseq combined with *in situ* data, suggests that Opa is competing with Oc for the regulation of a subgroup of genes later in gastrulation. Specifically, we find that Oc binds to late, head-specific enhancers independently and activates them in a head-specific wave of zygotic transcription, suggesting distinct roles for Oc in the blastula and gastrula stages.

## Introduction

Cell-type specification and differentiation occur early in embryonic development, and the core network of transcription factors (TFs) that lead to organogenesis are conserved in evolution. Pre-gastrulation developmental pathways have the greatest potential impact on development and disease as they precede and propagate those which follow (Farrell and O’farrell, 2014; Wamaitha and Niakan, 2018; Johnston and Nüsslein-Volhard, 1992). TFs which share homology and function between *Drosophila* and mammals present optimal utility in studying developmental phenomena with both broad and specific impacts, e.g., procephalic brain development (Finkelstein et al., 1990; Younossi-Hartenstein et al., 1997; Bridi et al., 2019) and the impact of those cell differentiation pathways and environmental factors on complex neurological disorders like autism spectrum disorder (ASD) (El Hayek et al., 2020).

Early embryos undergo multiple waves of zygotic genome activation regulated by well-orchestrated TF networks that lead to organogenesis (Briscoe and Small, 2015; Kwasnieski et al., 2019; Koromila et al., 2020). The roles of *Drosophila* embryonic transcriptional activators such as Bicoid (Bcd, PITX2 human ortholog) (Yoshioka et al., 1998), Zelda (Zld, POU5F1 human ortholog) (Yamada et al., 2019), Odd-paired (Opa, zinc finger protein of the cerebellum 3 (ZIC3), human ortholog) (Purandare et al., 2002), and Ocelliless (Oc, also known as Orthodenticle (Otd), OTX2 human ortholog) (Montalta-He et al., 2002), are largely conserved across animals (Matsuda, 2017) making them attractive targets for investigating broad species developmental and disease mechanisms in this well-established model organism. Gene replacement experiments show that the *Drosophila oc* gene and orthologous mammalian *Otx2* gene are functionally equivalent (Leuzinger et al., 1998; Montalta-He et al., 2002; Acampora et al., 2009; Terrell et al., 2012). In head development, different levels of OTX protein are required for the formation of specific subdomains of the adult head (Acampora et al., 2000; Acampora et al., 2009). Also, ZIC2, has been known to play major roles in neural progenitors regulation (Inoue et al., 2007; Iida et al., 2020). The critical nodes of the regulatory networks are promoter regions which are required for gene transcription; however, a significant part of transcriptional regulation occurs *via* the action of multiple cis-regulatory modules, enhancers, where TFs bind in various combinations to activate or repress target genes (Koromila and Stathopoulos, 2017; Furlong and Levine, 2018; Koromila and Stathopoulos, 2019). Furthermore, a gene with a complex expression pattern may have several region-specific enhancers active at any particular stage, each responsible for a discrete spatiotemporal aspect of the gene’s expression. Most enhancers can act either simultaneously or in sequence to support gene expression at different developmental points (Dunipace et al., 2013; Ferraro et al., 2016; Koromila and Stathopoulos, 2017).

Within the first hour of *Drosophila* development, transcriptional regulation shifts from maternally loaded control to zygotic regulation (Maternal to Zygotic Transition; MZT) (Harrison et al., 2011; Yamada et al., 2019). The ubiquitous TF Zelda that opens chromatin at enhancer regions during MZT, and allows initiation of zygotic gene expression (Sun et al., 2015), is followed by a late expressed pioneer-like factor, Opa. Specifically, there is a hand-off from Zld to Opa in zygotic genome activation at cellularization (Koromila et al., 2020; Soluri et al., 2020). Additionally, Bcd can bind to inaccessible chromatin on its own at high concentrations anteriorly (Chen et al., 2012; Mir et al., 2017; Hannon et al., 2017; Huang et al., 2017), but requires input from Zld and possibly other uncharacterized factors at low concentrations (Mir et al., 2017). Datta *et al.* previously showed that a group of Bcd-bound Anterior-Posterior axis (AP) enhancers are initially activated by Bcd, and later activation is transferred to Oc *via* a feed-forward relay (Datta et al., 2018). In the same study the authors described other head-specific enhancers that require other than Bcd factors for activation (Datta et al., 2018).

The broadly-expressed late-acting TF Opa drives the transcriptional landscape to undergo a dramatic shift to prepare the syncytial nuclei for cellular sovereignty rounding out the blastula stage and transitioning the embryo into gastrulation (Hursh and Stultz, 2018). Opa and Oc begin their expression at stage 5 with known thorax and head developmental functions, respectively (Acampora et al., 2000; Tao and Schulz, 2007). Super-resolution microscopy revealed that Opa and Oc are transiently coexpressed in a small region proximal to later formed cephalic furrow during cellularization. However, the differential action between the pioneer factor Opa and Oc on epigenetic timing and levels of gene expression in the embryonic head is still unknown.

A vast and growing number of genomics and transcriptomics studies have produced a panoply of ChIPseq, whole embryo and single-cell RNAseq (scRNAseq) (Calderon et al., 2022), and other genomics datasets available to the public. Our *in vivo* data were compared to these public datasets to reveal mechanisms of transcriptional control otherwise undetectable. We found that balance between a pioneer factor (Opa) and a localized activator (Oc) is important in regulating timing of gene expression pre-gastrulation. Further, meta-analysis of scRNAseq data reveals *opa/oc* coexpressing cells at stage 5 and enrichment of several known neural developmental genes in cells containing both *opa* and *oc* transcripts. Interrogation of these genes against Opa, Oc, Bcd, and Zld ChIPseq datasets, RNA expression databases, and published enhancer data suggests that Opa acts together with Oc for the regulation of a subgroup of head-specific genes, in both AP and Dorsal-Ventral (DV) axes, before gastrulation. Also, this study showed that Oc regulates head-specific Bcd/Opa-independent enhancers during gastrulation at a new cell-specific wave of zygotic activation. This is a powerful system to understanding head-specific gene activation in the early embryo.

## Results

### Opa and Oc co-occupy genomic loci and embryonic region pre-gastrulation

**FIGURE 1:**
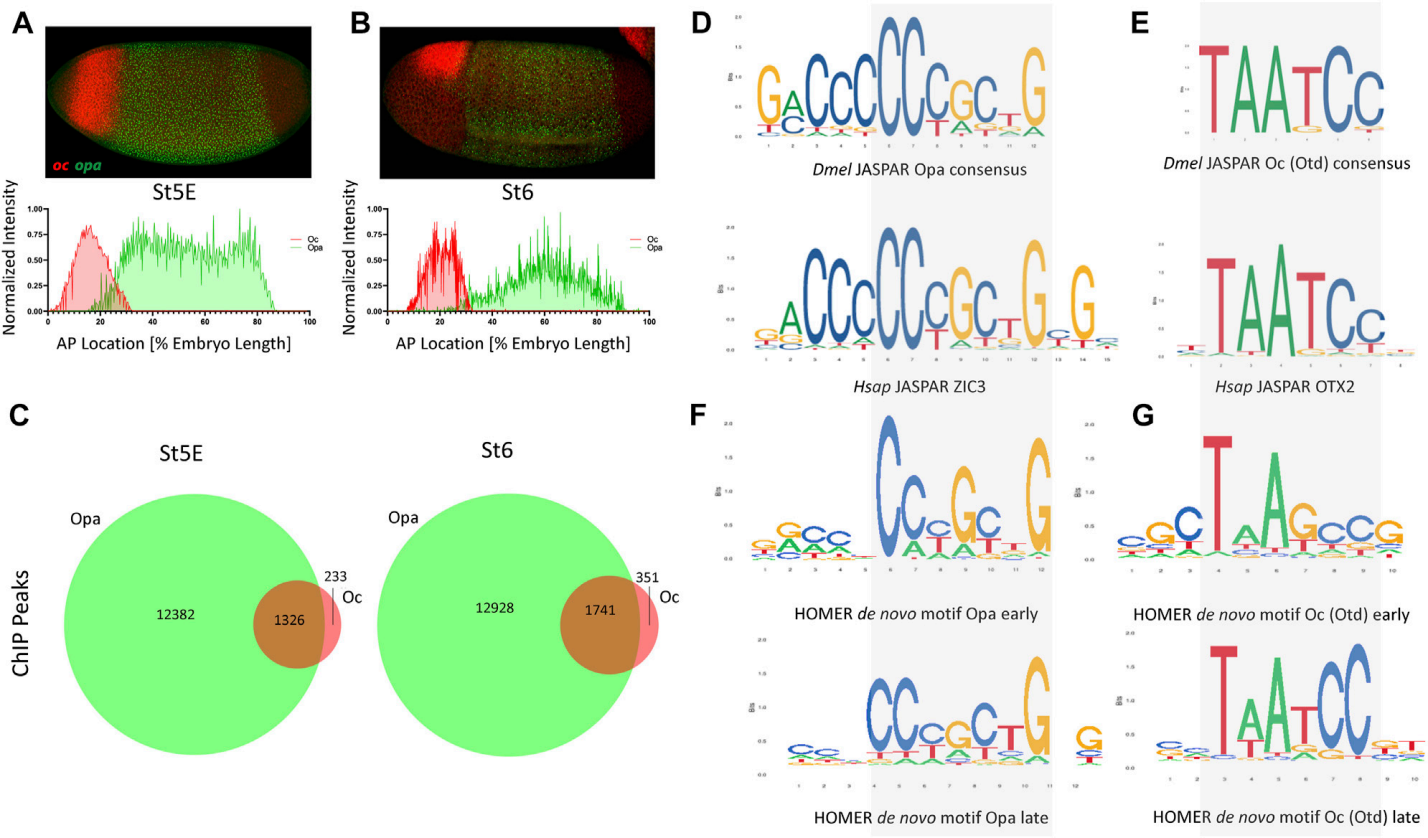
*opa* and *oc* dynamic overlap within the developing embryo. **(A)** At nc14B, a clear overlap between *oc* and *opa* domains can be observed (above) as is visualized graphically by batch plotting of AP FISH image fluorescence intensity below (n = 4,3). **(B)** By initiation of gastrulation, *oc* and *opa* expression domains become nearly distinct, as verified by batch plotting of AP FISH image fluorescence intensity (n = 7,7). **(C)** Venn diagrams representing ChIP peaks for Opa (green) and Oc (red) at early and late stages. (*p* <.0001, χ^2^) **(D–E)** Consensus binding data from JASPAR show that the *Drosophila* (*Dmel*) Opa **(D)**, and Oc **(E)** consensus binding sites are conserved in Human (Hsap). **(F–G)** Homer *de novo* motifs for Opa **(F)** and Oc **(G)** for early and late stages.

We first sought to investigate the expression dynamics of *opa* and *oc* at 4 time points, just before cellularization (Stage 5 early: nc14B), at two points during cellularization (Stage 5 late: nc14C and nc14D) and at the onset of gastrulation (Stage 6). At stage 5 early (St5E), we found that both *oc* (anterior, future head, region of the embryo) and *opa* (broad trunk region of the embryo) are expressed as previously described Figure 1A. Further investigation revealed that upon commencement of their transcription, *oc’s* and *opa’s* expression domains overlap in the posterior portion of the future head region (Figure 1A). This overlapping domain remains through cellularization but shrinks as cellularization ends and gastrulation begins (Figures 1A,B, Supplementary Figures S1A,B,C) (Sandler and Stathopoulos, 2016). Quantitative analysis of normalized fluorescent signal reveals an apparent posterior shift of the anterior boundaries of both *oc* (4%–8%) and *opa* (16%–25%) expression domains with the overlapping region shrinking from around 16%–7% of the AP body axis between St5E and initiation of gastrulation (Figures 1A,B). To investigate whether Opa and Oc may cooperate to affect gene expression in this overlapping region, we interrogated publicly available, whole embryo ChIPseq data for Opa and Oc genomic binding (Datta et al., 2018; Koromila et al., 2020). Intriguingly, we found that a large majority of Oc ChIPseq peaks overlap with Opa peaks with 85% of Oc peaks during early cellularization and 83% of Oc peaks at the onset of gastrulation coinciding with Opa peaks (Figure 1C).

The existence of a shrinking overlap in expression of *opa*/*oc* implies existence of a narrow spatiotemporal window where these TFs may be capable of simultaneous occupation of enhancer regions in a small group of early embryonic cells to initiate a transient, dynamic lineage, whilst maternal TFs, e.g., Bcd and Zld, phase out through cellularization in favor of zygotically expressed TFs (Supplementary Figure S1D). Using the peaks of these ChIPseq datasets to identify potential consensus binding sites, we next performed *de novo* motif analysis on these datasets to confirm consensus preservation between these datasets and the published, evolutionarily conserved JASPAR (Khan et al., 2018) motifs for Opa/ZIC3 and Otd/OTX2 (Figures 1D–G). Interestingly, *de novo* motif analyses for Opa and Oc (Figures 1F,G), while clearly resembling the JASPAR motifs, more precise motifs for St5L than St5E suggesting potential binding site competition early. Specifically, 200 bp regions centered at these ChIP peaks were analyzed using the HOMER program to identify overrepresented sequences that align to binding motifs. At stage 5E, a 10 bp core sequence with homology to the 6 bp Oc JASPAR consensus (Figure 1G, compare with 1E) was present in over 18.41% of all peaks. A second 10 bp motif exhibiting extended homology with the JASPAR Opa consensus was also identified through analysis of the Oc stage 5E ChIP-seq dataset, but this extended site is present at lower abundance (3.88%). However, there is a notable mismatch in the middle of the core sequence; while the *de novo* Oc consensus from the stage 5E ChIP-seq dataset does not include thymine at this position, both the JASPAR motif and *de novo* Oc consensus derived from the stage 6 ChIP-seq dataset (present in over 16.16% of all peaks) do (Figures 1E, G; bottom motif). These sequence discrepancies may relate to differences in optimal affinities for binding sites at different stages of development.

### Opa and Oc binding resolves post-cellularization

As we hypothesized distinct and cooperative roles for Opa and Oc TFs during cellularization, we next wished to investigate the binding dynamics of Opa and Oc during the mid-blastula transition (MBT). Toward this end, we further interrogated our HOMER *de novo* motif analyses from St5E and St6 embryos.

**FIGURE 2:**
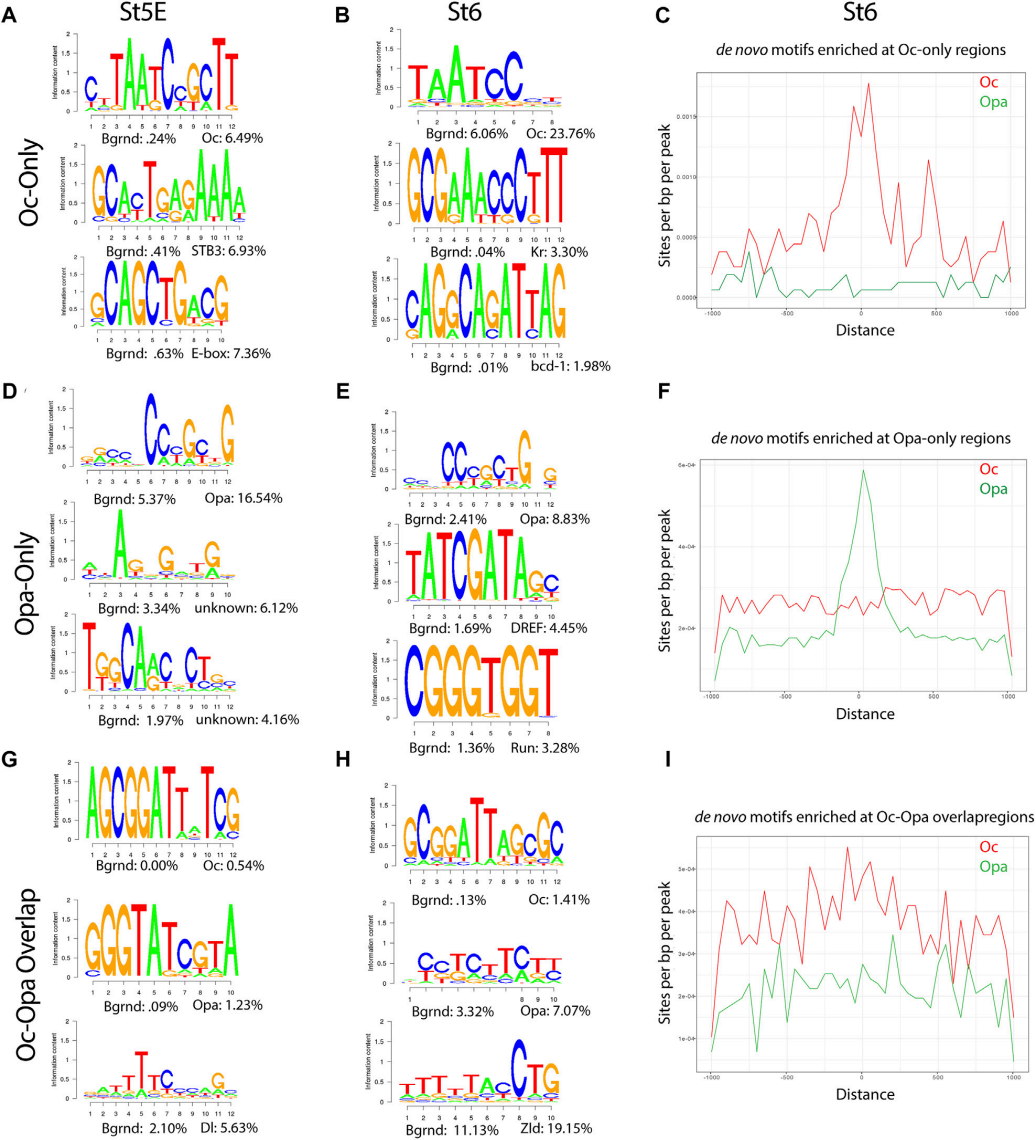
Enrichment of Opa and Oc *de novo* motifs in subsets of peaks that correspond to Opa-only, Oc-only, or Opa/Oc-bound regions identified by ChIPseq **(A,D)** and **(G)** Stage 5E HOMER *de novo* motif analyses for Oc-only (no Opa) **(A)**, Opa-only (no Oc) **(D)**, and Opa and Oc-only **(G)** peaks. **(B,E)** and **(H)** Stage 6 HOMER *de novo* motif analyses for Oc-only (no Opa) **(B)**, Opa-only (no Oc) **(E)**, Opa- and Oc-only **(H)** peaks. **(C,F)** and **(I)** Enrichment plots of Stage 6 Oc-only **(C)**, Opa-only **(F)**, and Oc-Opa overlapping peak **(I)** motifs at Oc (red) or Opa- and Oc-only (green) (Distance in bp).

Motifs from *de novo* analyses of Oc and Opa non-overlapping peaks matched the characteristic motifs for each TF, TAATCC and CCCGCTG, respectively in both early and late datasets (Figures 2A,B,D,E). As expected, when the ratio of these TF to maternal factors and total peak counts were low, the early dataset did not produce meaningful aggregations of predicted motifs around Oc peak loci (Supplementary Figure S2.1B). However, at the onset of gastrulation, there are significant peaks to reveal aggregation of Oc and Opa motifs at sites of their respective ChIPseq peaks (Figures 2C,F). Motifs from *de novo* analysis of Oc and Opa overlapping peaks also included the characteristic motifs for each TF in both early and late datasets (Figures 2G,H). Interestingly, there appears to exist a shift in Oc and Opa motif proximity to their individual ChIPseq peaks from clustering more around Opa peaks early to clustering more around Oc peaks at the later stage (Figure 2I and Supplementary Figure S2).

To further investigate Oc and Opa binding dynamics we compared the same predicted motifs against only those peaks from the previous analyses which did not overlap with Zld peaks. Removing Zld overlapping peaks produced negligible changes early, but resulted in a marked coalescence of Oc-only motifs around the remaining Oc peaks (Supplementary Figure S2.2A–F) demonstrating lower Oc motif density at Zld-binding loci at the late stage (St6).

A feed forward relay from Bcd to Oc has previously been shown (Datta et al., 2018), so we performed *de novo* motif analysis on publicly available Bcd ChIPseq data to compare to the results of the Oc motif analysis at St5E (total and Oc-only peaks), when the two factors are most co-expressed (Figure 2A, Supplementary Figure S2.1A and Supplementary Figure S2.2G). The predicted motifs are highly similar “Oc-only and Bcd at St5E”, further supporting the published concept of a Bcd-to-Oc hand-off (Datta et al., 2018). We further performed *de novo* motif analysis of those Oc peaks which do not overlap with Bcd peaks and did not find any major differences to the Oc-only motif analysis (Figure 1G, Supplemetary Figure S2.2H).

### Opa and Oc overlap in a narrow temporal window during embryogenesis

**FIGURE 3:**
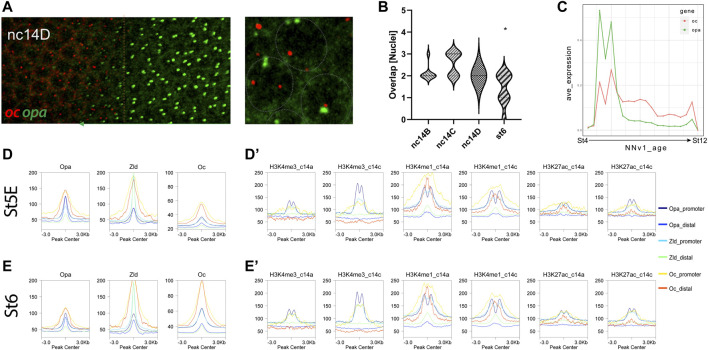
Opa/Oc subcellular protein and transcriptional dynamics. **(A)** Super resolution microscopy reveals simultaneous transcription of *opa* (green arrow region) and *oc* (bounded by orange dotted line) within individual nuclei (grey dotted lines) in the *opa/oc* overlap region. **(B)** Nuclei counts of *opa/oc* overlap region width by stage from the onset of *opa* expression to St6. The overlapping region is significantly lower at St6 than any of the cellularization stages (*p* <.05 compared to all other individual stages) (nc14b: n = 8, nc14C: n = 7, nc14D: n = 3, St6: n = 8). **(C)** Neural network age prediction plot of *oc* and *opa* expression from publicly available scRNAseq datasets (Karaiskos et al., 2017; Calderon et al., 2022). X-axis values are neural network age prediction pseudotimes between stage 4 and 12 **(D, E)** Stage 5E **(D)** and Stage 6 **(E)** Opa, Zld and Oc ChIPseq peak correlation by promoter or distal enhancer subclusters, as indicated in the key. **(D′, E′)** Stage 5E **(D′)** and Stage 6 **(E′)** H3K4me3, H3K4me1 and H3K27ac at nc14a and at nc14C signal intensities centered at different ChIP-seq regions (promoters or distal enhancers). For the two different timepoints nc14A and nc14C, different Zld and Oc ChIP data were used, as indicated in methods.

Having determined that the *opa* and *oc* expression domains overlap, we sought to confirm that this overlap results in expression of both TFs within individual cells in the overlapping region. Using super resolution microscopy we were able to image individual allele transcription of both *oc* and *opa* within individual nuclei of the overlap region (Figure 3A). We were further able to confirm that the overlapping region diminishes as cellularization ends and gastrulation begins by counting cells along the AP axis coexpressing both transcripts (Figure 3B). This finding is intriguing as this overlapping domain resides in the procephalic region of the embryo which will eventually beget the nascent brain and implies the potential for dual binding of these two activators uniquely within these cells.

To better characterize the temporal dynamics of *opa/oc* expression overlap, we analyzed publicly available scRNAseq data spanning 1–7h into embryonic development (∼St4-12) (Supplementary Figure S3.1A–E). We further used neural network age prediction of the transcriptomic temporal landscape (Calderon et al., 2022) to visualize *opa/oc* expression (Supplementary Figure S3.1B–E). In support of our findings that the *opa/oc* overlapping region is transient, we found that *opa* and *oc* expression peaks at approximately the cellularization/gastrulation transition (Figure 3C). Intriguingly, we also found that *opa* and *oc* expression drops early in gastrulation as well (Figure 3C). *oc* diminishes much more gradually than *opa* and a small population (45 cells (Calderon et al., 2022)) of *opa/oc* coexpressing cells arises transiently between 1 and 3h post fertilization (Figure 3C).

To examine the potential for Opa and Oc cooperation during this stage of development, we compared St5 chromatin occupancy of Opa and Oc between gene loci based on their published expression shifts following *opa* knockdown (KD) (Datta et al., 2018; Koromila et al., 2020). Oc-only peaks reside predominantly at gene loci insignificantly changed by *opa* KD as do Opa peaks (Supplementary Figure S3.1F). However, Oc peaks broadly correlate most strongly with gene loci of genes which increase in expression following *opa* KD contrary to Opa-only peaks which associate most strongly with insignificantly affected gene loci (Supplementary Figure S3.1F,G). Intriguingly, together, this suggests that a small number of genes may be conversely regulated by Opa and Oc.

We next sought to investigate potential differences in Opa and Oc peak genomic distributions. Both TFs correlate with promoter marker, H3K4me3, early and late peak locations similarly between St5E and St6 at gene promoters, but not enhancers (Figures 3D’, E’, Supplementary Figure S3.2G,H). Further, both TFs bind similarly between stages 5E and 6 to genomic loci marked by poised-transcription marker, H3K4me1, regardless of whether the loci were marked prior to, after, or during the peak binding at promoters (Figures 3D’, E’). Interestingly, however, Oc binding at distal enhancers also clusters around H3K4me1 marks while only promoter binding sites for either Opa or Oc cluster at transcriptional activity marker, H3K27ac (Figures 3D’, E’, Supplementary Figure S3.2G,H). Curiously, at promoter loci, Oc and Opa appear to occupy complimentary niches relative to these histone marks, with Opa binding coalescing bimodally around the histone marks and Oc peaks centering atop them, reflecting a more Zld-like profile for Oc than Opa (Figures 3D,E, Supplementary Figure S3.2). Strikingly, Oc promoter and distal enhancer peaks increase dramatically between St5E and St6 at Zld peak loci (Figures 3D,E, Supplementary Figure S3.2).

### Opa and Oc overlap is likely involved in spatiotemporal localization of downstream AP and DV gene expression in that region

**FIGURE 4:**
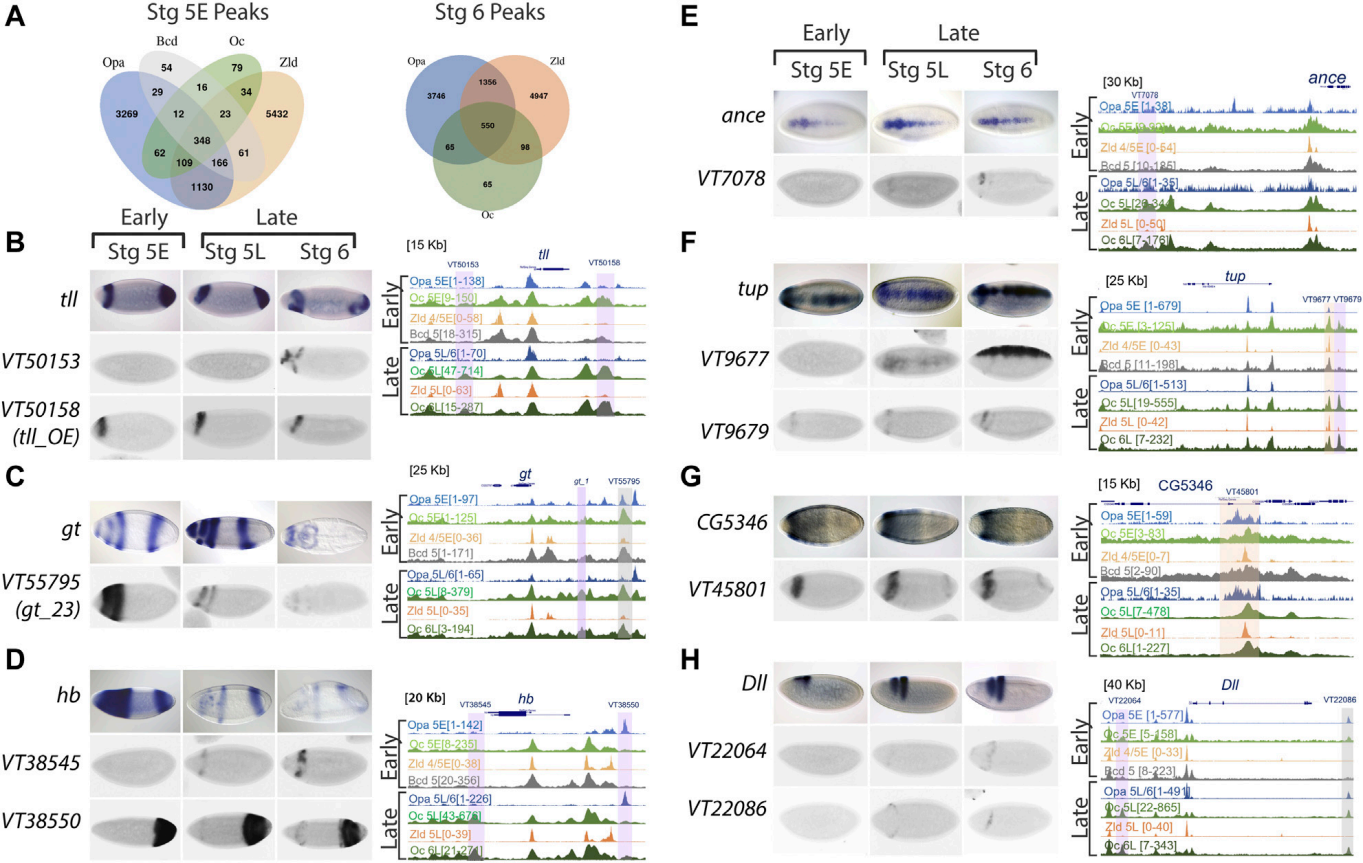
Oc ChIPseq data demonstrates binding in both AP and DV axis, including some stage 5 enhancers, as well as other later acting enhancers. **(A)** Venn diagrams of Opa, Oc, Bcd, and Zld peaks at distal enhancers at stages 5E and 6. **(B–H)** ChIPseq data and enhancer expression patterns for important developmental factors. ChIPseq datasets: Opa at stage 5 early (light blue), Oc at stage 5 early (light green), Zld at stage 4/5 early (light orange), Bcd at stage 5 (grey), Opa at stage 5 late/stage 6 (dark blue), Oc at stage 5 late (olive green), Zld at stage 5 late (dark orange), and Oc at stage 6 late (dark green) from previous studies were aligned using UCSC genome browser. Numbers in square brackets indicate maximal peak heights and colored highlights marking the peaks indicate enhancers of interest with different occupancy in our study. The grey highlights mark Oc/Opa binding, light purple indicates Opa or Oc individually bound regions, and orange highlights mark Zld-bound enhancers. For each panel **(B–H)**, endogenous expression patterns for **(B)** *tailless* (*tll*), **(C)** *giant* (*gt*), **(D)** *hunchback* (*hb*), **(E)** *angiotensin converting enzyme* (*ance*), **(F)** *tailup* (*tup*), **(G)** *CG5346*, and **(H)** *distal-less* (*Dll*) are extracted from publicly available Fruitfly database, and expression patterns of highlighted enhancers (Vienna tile ID in blue above).

Further, to interrogate the regulatory dynamics of Opa and Oc, we looked at peak overlap between Opa, Oc, Bcd, and Zld. Nearly all Oc peaks overlap with at least Opa or Zld at both St5E and St6 (89% early, 96% late; Figure 4A). Interestingly, there is a large difference between distal enhancer and promoter loci in this regard wherein Oc-only peaks are more than twice as frequent in distal enhancers than promoters (Supplementary Figure S4, Figure 4K–N). Additionally, this reduction in Oc-only peaks at promoters seems to be nearly entirely driven by overlap, especially St6 overlap, with Zld peaks. Together these data suggest that Oc may play a more independent role at distal enhancers than promoters where Oc, similar to Opa (Datta et al., 2018; Koromila et al., 2020), may be acting as a regulatory substitute for waning Bcd and Zld factors.

We next explored enhancer occupation by Opa, Oc, Bcd, and Zld at targets with distinct expression near the Opa/Oc overlap domain or known to play major roles in brain and neuroblast development. We found that Oc and/or Opa occupy enhancers near *giant (gt), tailless (tll), hunchback (hb), distal-less (Dll), Angiotensin converting enzyme (ance), tailup (tup), eyeless (ey), CG5346, empty spiracles (ems), buttonhead (btd), toy, H6-like-homeobox (hmx), disheveled (dsh), lysine demethylase 5 (kdm5), eyeless (ey), ventral nervous system defective (vnd), posterior sex combs (psc),* and *acheate (ac)* (Figures 4B–H and Supplementary Figure S4A–J). Those Oc and/or Opa occupied enhancers at these genes with archived gene trap expression patterns in the StarkLab database (Kvon et al., 2014) are characterized by a clear pattern of expression at or near the Opa/Oc overlap domain (Figures 4B–H). Intriguingly, several genes being regulated in the overlap region are expressed in a DV pattern suggesting this Opa/Oc co-regulation is not limited to AP patterning (Figures 4E–H).

**FIGURE 5:**
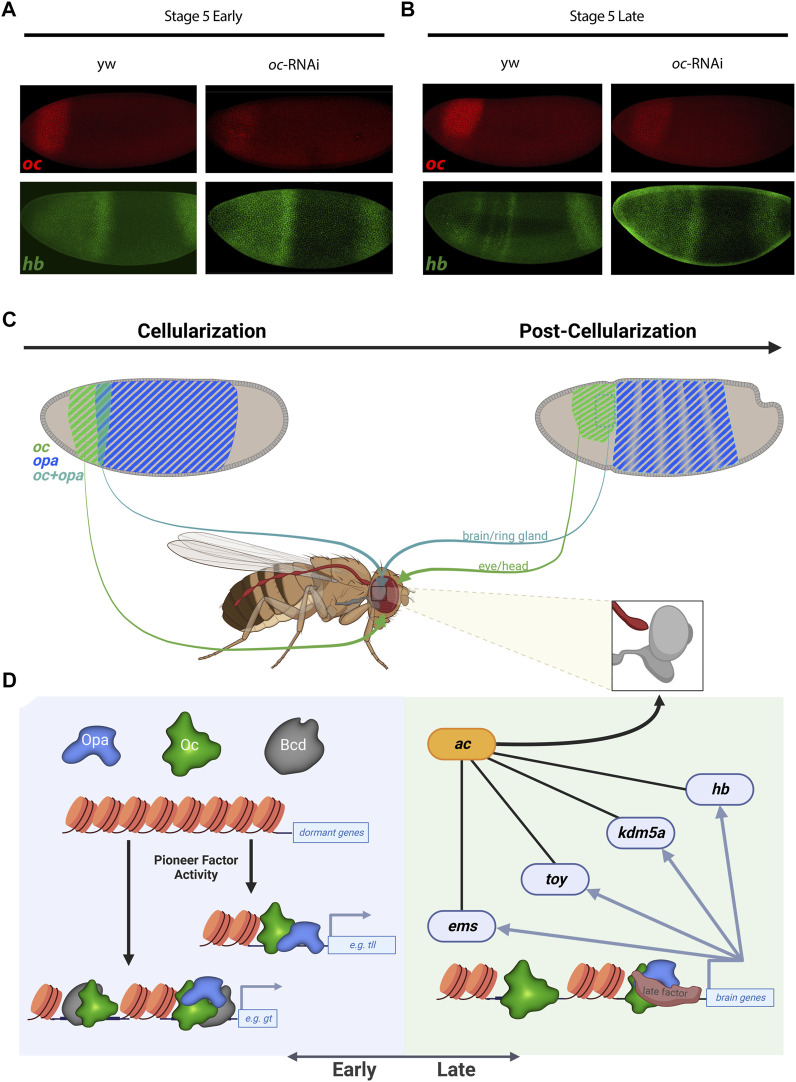
Oc and Opa play diverse roles in embryonic head development. **(A)** and **(B)** *oc* depletion by *shRNAi* results in an failure of late second *hb* band to resolve from broad early expression when Oc ChIPseq shows Oc occupancy, complimenting the V38545 enhancer expression pattern shown in Figure 4D. (wt: Early: n = 4, Late: n = 3 |*oc-RNAi*: Early: n = 5, Late: n = 3) **(C)** and **(D)** Model illustration: Overlapping *opa* and *oc* expression generates a progenitor pool during cellularization (**C**, Left) which eventually begets the brain **(C)**. At the genome level, early transcription factors bind promoters and enhancers to switch on genes, fascilitated by the pioneer factor activity of TFs like Opa and Bcd (**D**, Left). Later during embryonic development, pioneer factor activity is no longer required and independent roles for Oc and downstream TFs drive brain development (D, right). **(C,D)** partially created with BioRender.com).

To test these analyses of Opa and/or Oc target genes *in vivo*, we sought to experimentally reproduce a result from our analyses. Using *oc shRNAi* (Kim et al., 2004) embryos to knock down Oc levels, we were able to eliminate, at St5L when Opa and Oc are no longer simultaneously available to potentially compensate for one another, early pattern, located in the region of *opa/oc* co-expression, of anterior *hb* expression (Figures 5A,B). Intriguingly, a band at this location can be reproduced exogenously *via* enhancer-driven *lacZ* as observed from the StarkLab database (see Figure 4D). Together, these data suggest dynamic roles for Opa and Oc in gene regulation which include a clear potential for establishment of a head lineage niche beginning within their early, transient overlapping region.

## Discussion

In this study, we combine *in vivo* experimentation and meta-analyses to reveal Opa/Oc epigenetic dynamics during early embryonic development. Oc is a late-acting timing factor which regulates head gene expression in the embryo, along DV as well as AP axes. A transient overlap in Opa and Oc expression in cells likely destined for brain development before the onset of gastrulation, led us to investigate Opa’s role in head development for the first time. During the short period of overlap (nc14B-nc14D), we noticed that both Opa and Oc binding sites on the genome are less resolved than after their expression domains diverge suggesting the possibility of cooperative and/or competitive binding between the two factors at different time points. This implication was further supported by our finding that a broad majority of Opa and Oc peaks overlap on the genome. Additionally, gene ontology analysis of the genes occupied by Opa and/or Oc reveals several neurogenic processes among the top hits for genes occupied by both TFs (Supplementary Figure S1E). Together, these early findings strongly point toward a developmental instrument for the specification of a cell primordium concurrent with or preceding the onset of gastrulation.

Interestingly, we found association of both Opa and Oc ChIPseq peaks with late pioneer and chromatin architecture factors, such as Trl/Gaf and Dref-1/Beaf-32 binding motifs (Heinz et al., 2010). However, inspection of publicly available ChIPseq data (Gaskill et al., 2021) revealed that Gaf does not appear to bind at head-specific Opa/Oc-bound enhancers investigated in this study (data not shown). Further investigation is needed to untangle the intriguing implications of this finding and to determine whether Opa or Oc peaks are involved in topologically associated domain (TAD) insulator functions. Through investigation of ChIPseq datasets, we were further able to identify relative shifts in Opa and Oc binding to Zld peaks at promoters rather than distal enhancers, supporting a model whereby Opa and Oc regulate transcription at late enhancer regions (Supplementary Figure S4K–N). However, further investigation is needed to determine whether sequential binding of the two proteins to the early identified DNA motif is related to cell specification, and whether Oc activates a late head-specific wave of zygotic transcription in brain cells via binding to the late, Bcd-like, motif.

Despite the broad and abundant genomic occupancy by Opa, the expression of some genes remains unchanged when Opa protein is diminished (Koromila et al., 2020), implying cooperative and compensatory transcriptional regulation with other TFs of similar regional spatiotemporal abundance, such as Oc. The expression patterns of both *opa* and *oc* are very dynamic at stage 5. We characterize the transient *oc/opa* overlap, using regular confocal and super resolution microscopy coupled with both manual and automated quantification techniques. scRNAseq meta-analysis also confirmed the dynamic expression of the two genes in the embryo (Calderon et al., 2022). Much of the epigenetic landscape remains unexplained at the cellularization/gastrulation transition and these Opa/Oc dynamics are undoubtedly involved; future studies are needed to investigate how the embryo utilizes this unique cell niche to pattern the brain/head.

We can postulate about some of these epigenetic mechanisms by considering Opa and Oc TF binding relative to histone marks (Figure 3 and Supplementary Figure S3.2). We found that Oc, but not Opa, binding at loci distal to genic regions correlate with H3K4me1 histone mark indicative of enhancers or genomic regions poised for transcription initiation. In addition, Oc distal binding seems to somewhat localize around genomic regions which are transcriptionally active pre-cellularization indicative of the regulatory hand-off mentioned previously (Datta et al., 2018). Interestingly, Opa distal enhancer binding did not cluster around any of the histone marks tested. This likely stems from the previously reported “pioneer-like’ activity of Opa (Koromila et al., 2020) distal binding, a possibility which is further supported by the matching trend in Zld (Yamada et al., 2019). Together, a likely model is that maternal Bcd acts early and Opa acts late through the transient *opa/oc* overlap to facilitate Oc binding to distinct genomic loci, producing distinct fates in the anterior vs. posterior *oc* expression domain (Figures 5C, D).

We further investigated how Opa and Oc are associated and regulate the zygotic genome in both AP and DV axes at cellularization and at gastrulation. We found that these associations tend to correspond with binding at enhancer regions which drive expression in bands at or near the Opa/Oc overlapping domain. Rather than transcriptional activation being linked directly to absolute Oc concentration, Opa may act to modulate Oc’s effective concentration: e.g., lower levels of Oc may be required to activate enhancers bound by Opa. This model would explain our findings that some Oc-only peaks can drive narrow band expression in this region rather than across the entire *oc* expression domain and why knockdown of *oc* was sufficient to eliminate *hb* expression there as well. However, future work is needed to determine if this phenomenon is driven by Opa regulation of Oc levels, whether Opa plays a compensatory or cooperative role at Oc peaks, or some mixture of these and whether these regulatory dynamics are direct or indirect.

Future studies are needed to elucidate the mechanisms that give rise to the complex structures downstream of the Opa/Oc regulation investigated here. In particular, gain- and loss-of-function experiments to reveal the immediately downstream regulatory repertoire will be a valuable tool to eventually understand the process in this spatiotemporal pathway. Super resolution microscopy of nascent transcripts in live embryos (Hoppe and Ashe, 2021; Huang et al., 2023) coupled with future spatial genomics (Asp et al., 2020; Luo et al., 2022) studies are promising to more precisely elucidate Opa/Oc dynamics during this nascent transition. Additionally, we are hopeful that future experimental techniques will enable region-specific ChIPseq within the early embryo to overcome the occupancy resolution limitations inherent to whole embryo datasets.

Being evolutionarily conserved, the implication that Opa and Oc are pre-gastrulation cell specification factors is potentially relevant to higher species, including humans, both in the interrogation of nascent embryonic development and investigation of congenital disease, e.g., autism (El Hayek et al., 2020), epilepsy (Acampora et al., 2000; Montalta-He et al., 2002), and congenital heart disease (Ware et al., 2004).

## Methods

### Fly stocks and husbandry

Wild type flies used in this study were of the yw [67c23] strain. Flies were reared under normal conditions at 23°C, with the exception of short hairpin (*sh*) *RNAi* constructs crossed to Gal4 (Hales et al., 2015) and yw control flies for those experiments which were incubated at 26.5°C. Virgin *UAS-shRNA-oc (ocRNAi)* (Bloomington *Drosophila* Stock Center (BDGP) #34327, #29342) females were crossed to matzyg.Gal4 or MTD.Gal4 males (BDSC#, #31777). Depletion of oc was achieved by crossing the virgin females from this cross to *ocRNAi* males.

### *In situ* hybridization, imaging, and analysis

Standard protocols were used for 2–4h embryo collection, fixation, and staining. FISH was performed using antisense RNA probes labeled with digoxigenin-, biotin-, or DNP-UTP to detect transcription of target genes. UP-TORR (Hu et al., 2013) was used to confirm absence of off targets for *shRNAi* lines used. All *in vivo* experiments are with a minimum of 3 embryos per condition.

Images were acquired using a Zeiss LSM 900 “Airyscan 2” confocal microscope. Confocal images were taken using a 20x air lens and super resolution, Airyscan, images were taken using a ×40 water objective using 488nm, 561nm, and 647 nm lasers.

Image processing was performed in Fiji (ImageJ) using standard z-projection procedures. Processed images used for expression domain profiling were then used to create segmentation masks in Ilastik (Huang et al., 2017; Berg et al., 2019). To generate the expression domain plots, a Python script was used to average pixel fluorescent intensity for each channel in 10px-wide slices along the AP axis of the embryos; these data were then plotted in GraphPad Prism.

### Bioinformatics

Oc and Bcd ChIPseq bed peak files of dm3 coordinates were converted to dm6 using UCSC liftOver tool. Oc and Bcd ChIPseq bigwig signal traces were converted from dm3 to dm6 assembly using crossMap (v0.6.4, PMID: 24351709). Opa and Zld processed data (dm6 assembly) were collected from our previous study (PMID 32701060).

To understand overlapping of different transcription factor binding sites across the genome, peak regions were combined and overlapping peaks were merged. Combined regions that overlapped both Opa and Oc peaks were defined as Opa-Oc overlap regions; regions overlapping with either Opa or Oc peaks were defined as Opa-only and Oc-only regions respectively. Region overlap analysis was performed using bedtools (v2.30.0) and Venn diagrams were generated using VennDiagram R package. Further *de novo* motif analysis was performed on different ChIPseq regions using the HOMER program (PMID 20513432) with default parameters and with options -size 200 and -mask. Selected *de novo* motifs identified from peak regions were queried against the Opa-Oc overlap, Opa-only and Oc-only regions for comparison and for generating motif aggregation plots, with the -size 2000 -hist 50 options. DNA sequence logos were plotted using the seqLogo R package. ChIPseq peak regions were associated with nearest gene transcription start sites using the annotatePeaks.pl module of HOMER. Promoter peaks and distal peaks were distinguished using a distance cutoff of 3 kb to the nearest transcription start sites.

Opa-only, Oc-only, Opa & Oc overlap regions at early and late stages were annotated using HOMER program. Genes associated with the peak regions were extracted for gene ontology analysis using gprofiler2 package. Top10 terms enriched for individual regions were concatenated to generate a heatmap plot with color representing statistical significance (-log10FDR value).

In addition, computeMatrix and plotHeatmap modules of deepTools (v3.2.1) were used to calculate and plot normalized histone mark and transcription factor signal intensities surrounding selected ChIPseq regions. For this and all subsequent data presented using heatmaps, the first sample in the heatmap was used for sorting the genomic regions based on descending order of mean signal value per region; all other comparison samples were plotted using the same order determined by the first sample. UCSC Genome Browser (PMID 21221095) was used to visualize ChIPseq signals at individual loci.

ChIPseq peak-associated genes and RNAseq differentially expressed genes were subjected to overlapping statistical analysis (Fisher’s exact test), and the results were presented in overlap gene count and overlap *p*-value heatmaps.

Publicly available scRNAseq data was downloaded from GEO database (GSE190147). The processed gene count table of a total of 547,805 single nuclei from stages *Drosophila* embryos was subject to downstream analysis. As note, each single nucleus was assigned with a developmental age score (NNv1_age) using neural network-based prediction (Calderon et al., 2022).

To track gene expression across developmental stages, age score (NNv1_age) of each nucleus was rounded up to the nearest integer to calculate average gene count values for the nuclei at the same developmental stage (NNv1_age_bin). Gene expression values across 20 different time points were presented in a line plot (St4-12).

To explore co-expression of two genes at single cell level, double positive nuclei (at least 1 count for both genes) were separated from other nuclei. A violin plot was presented to show cell distribution across developmental age for both positive and negative groups.

Unless noted otherwise, R was used to calculate statistics and generate plots.

### ChIPseq procedure and analysis

ChIPseq was used to determine the binding sites of transcription factors and other chromatin-associated protein in the genome and to understand how proteins interact with the genome to regulate the gene expression in *Drosophila* embryo. ChIPseq libraries were generated from the University of California, Santa Cruz (UCSC) genome browser platform. The ChIPseq reads from previous studies were aligned were aligned to *Drosophila* reference genome assembly (UCSC dm3) (Datta et al., 2018) at different time points: Opa at stages 5 early and 5 late/stage 6, Oc at stages 5 early, 5 late, and 6 late, Zld at stage 5 early and 5 late, and Bcd at stage 5. The resulting alignment tracks helped us to detect important genomic regions to study the mentioned factors.

## Data Availability

The datasets presented in this study can be found in online repositories. The names of the repository/repositories and accession number(s) can be found in the article/Supplementary Material.
