# Supplementary material for: Cell-specific occupancy dynamics between the pioneer-like factor Opa/ZIC and Ocelliless/OTX regulate early head development in embryos
Source: Front Cell Dev Biol. 2023 Mar 27;11:1126507. doi: 10.3389/fcell.2023.1126507 (PMC10083704; doi:10.3389/fcell.2023.1126507)
Supplement: Supplementary file 1 [file DataSheet1.pdf]

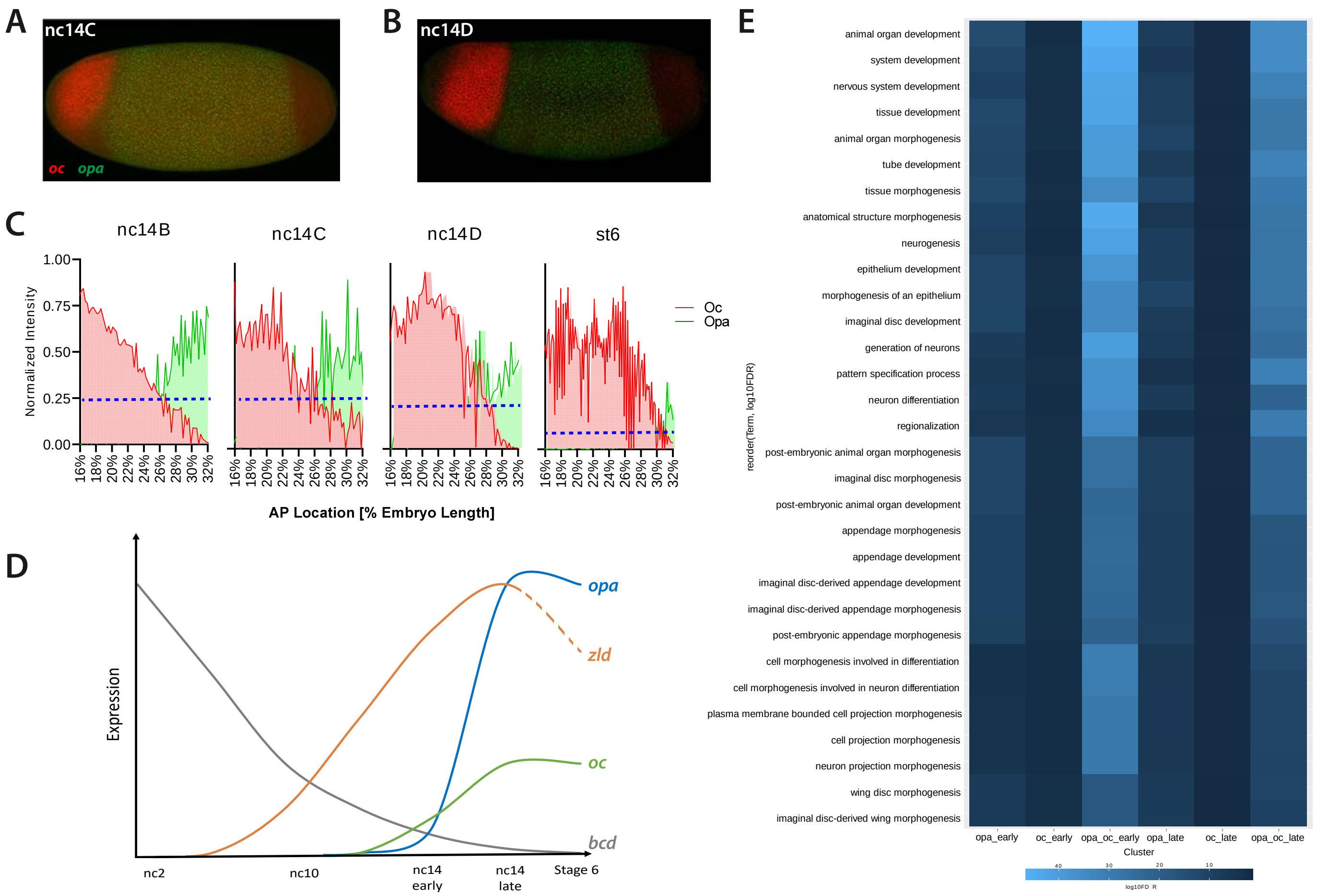

Supplemental Figure 1. Transcription factor dynamics during *Drosophila* embryo head development.

(A) opa and *oc* expression domains overlap within embryos as cellular segregation begins at nc14C. (B) As cellularization completes, the overlapping domain of *opa* and *oc* is visibly beginning to diminish. (C) Graphical batch plotting of AP FISH image fluorescence intensity analysis reveals reduction in *opa/oc* overlap as cellularization completes and gastrulation begins (blue dotted lines mark approximate relative levels of peak *opa/oc* overlap intensity). (D) The graph illustration reflects the expression level of genes *odd-paired (opa), zelda (zld), ocelliless (oc)* and *bicoid (bcd)* at different developmental stages, as stated in the figure. (E) GO analysis for genes occupied by Opa and/or Oc during Stage 5E (early) or Stage 6 (late).

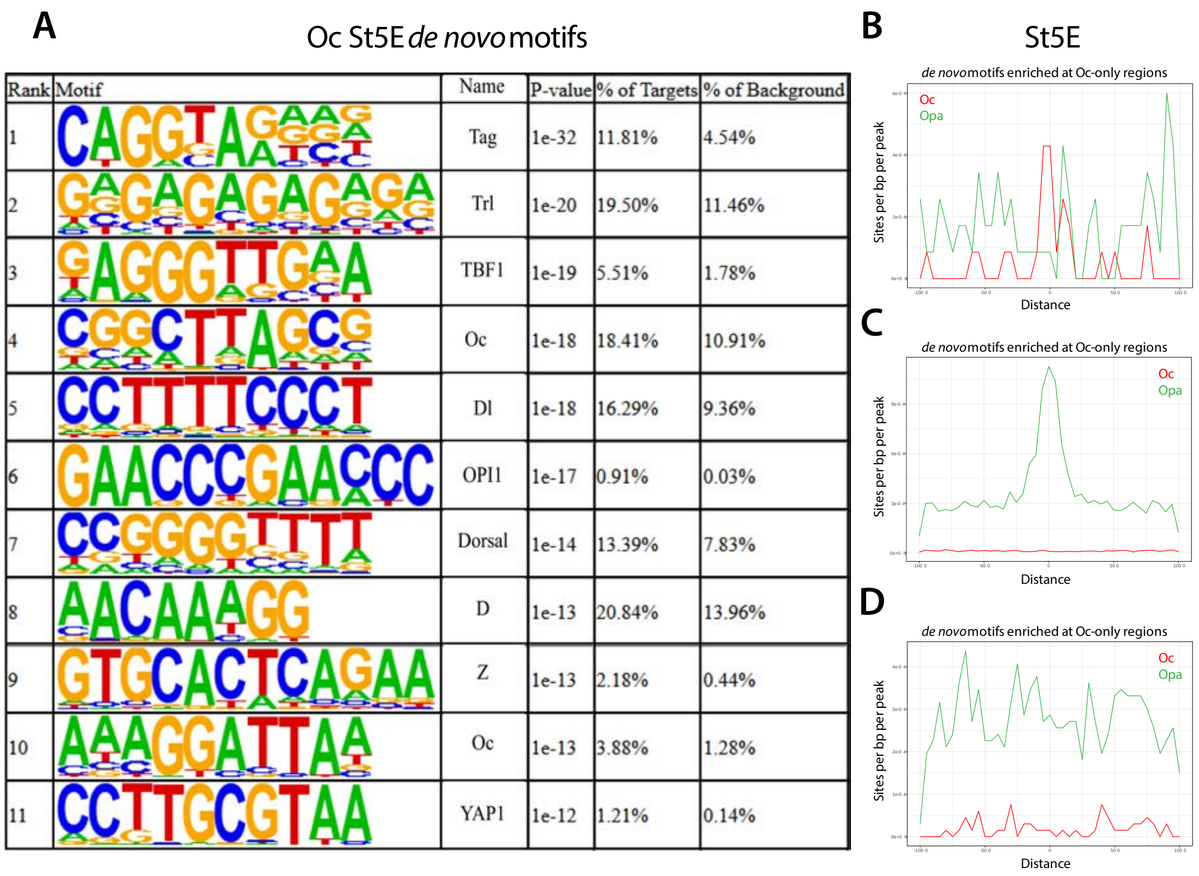

Supplemental Figure 2.1. Oc and Opa HOMER *de novo* motif analyses

(A) Table of stage 5E *de novo* predicted consensus motifs for Oc. (B-D) Stage 5E de novo motif enrichment plots comparing Oc or Opa peaks to the Oc only (B), Opa only (C), and Oc-Opa overlapping peak (D) motifs.

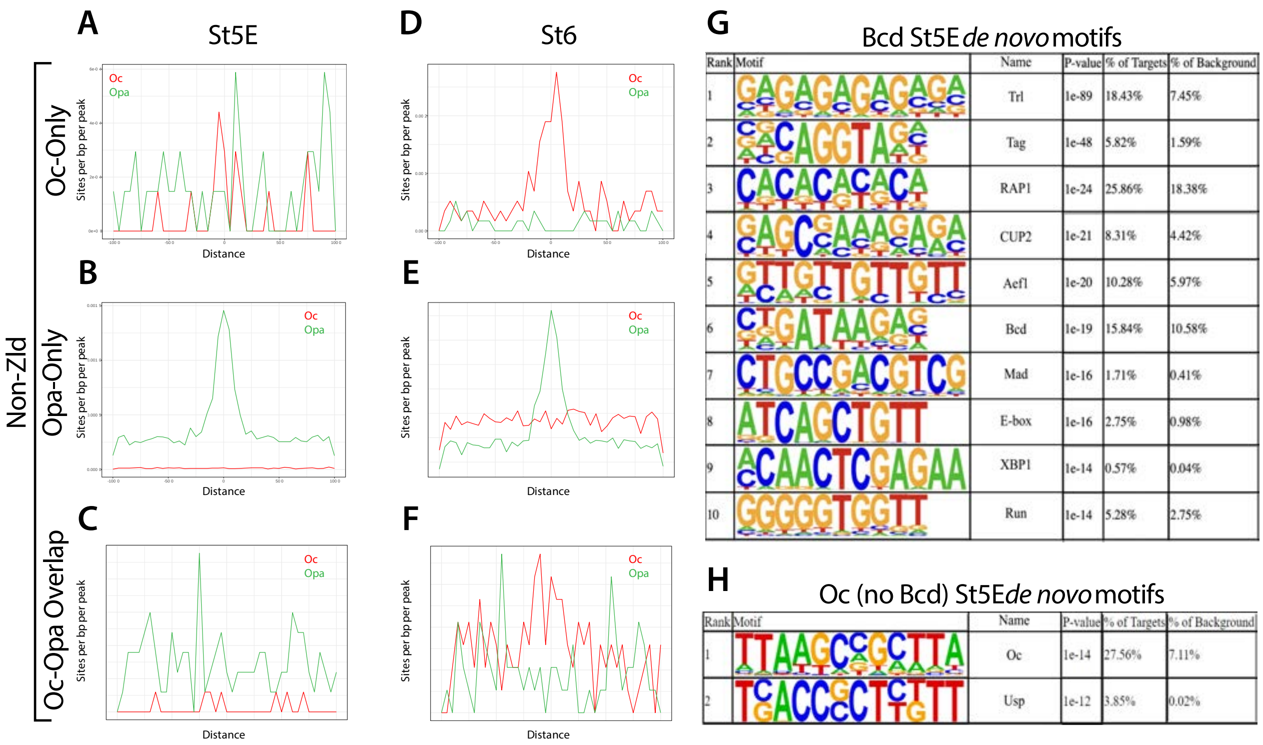

Supplemental Figure 2.2. Bcd, Oc, and Opa HOMER *de novo* motif analyses

(A-C) Stage 5E *de novo* motif enrichment plots comparing Oc or Opa peaks to the Oc only (no Opa/Zld) (A), Opa only (no Oc/Zld) (B), and Oc-Opa only (no Zld) overlapping peak (C) motifs. (D-F) Stage 6 *de novo* motif enrichment plots comparing Oc or Opa peaks to the Oc only (no Opa/Zld) (D), Opa only (no Oc/Zld) (E), and Oc-Opa only (no Zld) overlapping peak (F) motifs. (G) Table of stage 5E *de novo* predicted consensus motifs for Bcd. (H) Table of stage 5E *de novo* predicted consensus motifs for Oc peaks excluding those which overlap with Bcd peaks.

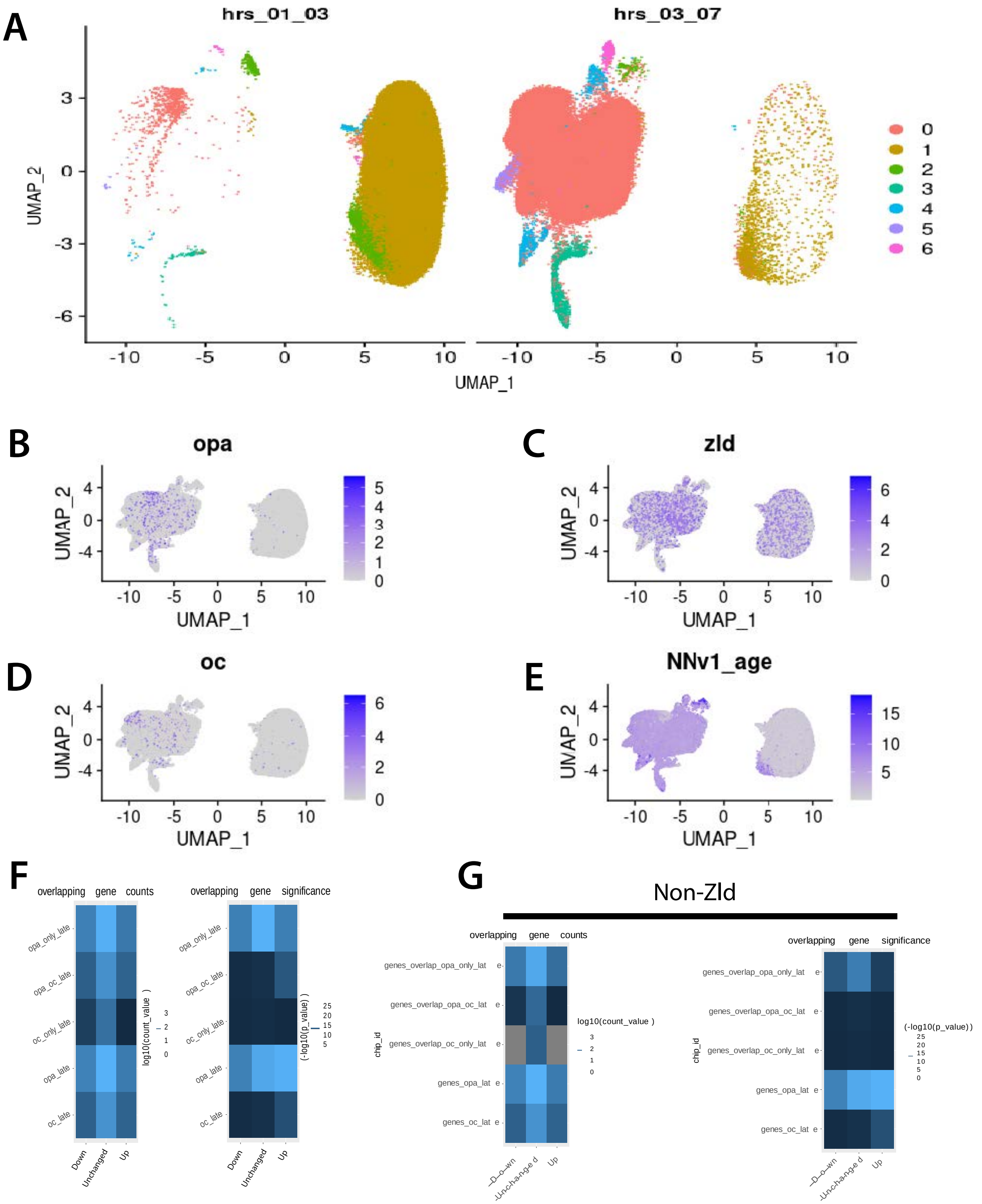

Supplemental Figure 3.1. Opa and Oc embryonic [4] expression and regulatory dynamics.

(A) UMAP cluster analysis of publicly available scRNAseq data from 1-3hr (~st4-6, left) and 6-11hr (st11, right) embryos. (B-D) UMAP plots showing *opa* (B), *zld* (C), and *oc* (D) relative cluster localizations. (E) Neural network age prediction analysis of UMAP data showing distribution of psuedotime ages spanning

from 0 to 20hr. (F) Opa and/or Oc occupation at gene loci either up regulated, down regulate, or unchanged from publicly available opa mutant RNAseq data by number of genes (left) and statistical correlation with genes (right). (G) Opa and/or Oc, but not Zld, peak localization at gene loci either up regulated, down regulate, or unchanged from publicly available opa mutant RNAseq data by number of genes (left) and statistical correlation with genes (right).

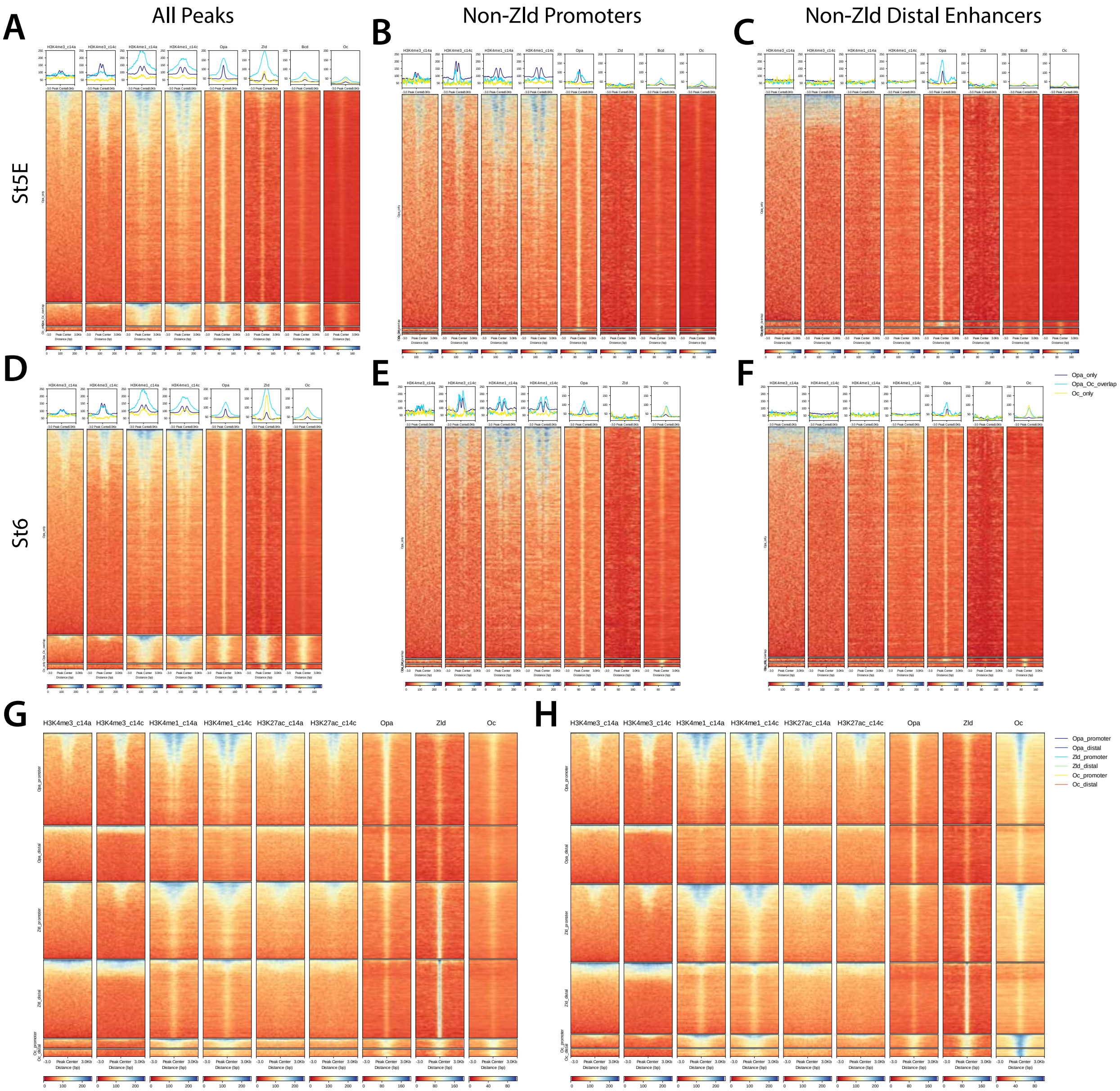

Supplemental Figure 3.2. Opa and Oc binding dynamics.

Stage 5E (A-C) and Stage 6 (D-E) Opa, Oc, and Zld ChIPseq peak correlation to common histone markers, Opa, Oc, Bcd, and Zld ChIPseq peak loci. (G,H) Heat maps for Stage 5E (G) and Stage 6 (H) Opa, Oc, and Zld ChIPseq peak correlation by promoter or distal enhancer subclusters to common histone marker, Opa, Oc, or Zld ChIPseq peak loci reported in Figure 3.

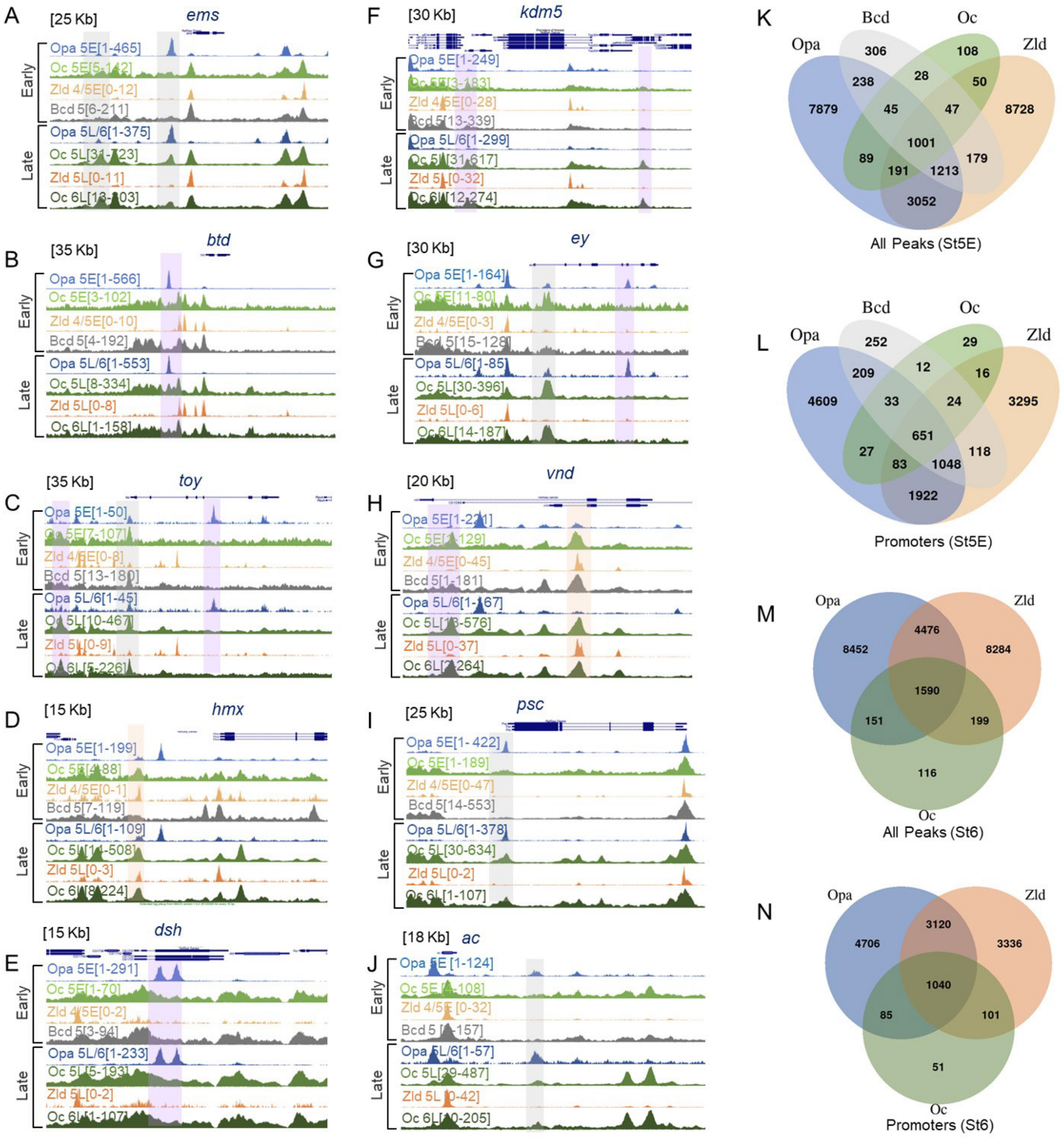

Supplemental Figure 4. Oc binds both AP and DV gene loci before and after gastrulation

(A-J) ChIPseq data indicating genome binding of Opa at stage 5 early (light blue), Oc at stage 5 early (light green), Zld at stage 4/5 early (light orange), Bcd at stage 5 (grey), Opa at stage 5 late/ stage 6 (dark blue), Oc at stage 5 late (olive green), Zld at stage 5 late (dark orange), and Oc at stage 6 late (dark green). Numbers in square brackets indicate maximal peak heights and colored highlights marking the

peaks indicate enhancers of interest with different occupancy in our study. Grey highlights mark Oc/Opa binding, light purple indicates Opa or Oc individually bound regions, and orange highlights mark Zld-bound enhancers. (K-L) Stage 5E Venn diagrams for Opa, Bcd, Oc and Zld total (K) and promoter only (L) ChIPseq peaks and for stage 6 Opa, Oc and Zld total (M) and promoter only (N) ChIPseq peaks.
